# An *in silico* model of the capturing of magnetic nanoparticles in tumour spheroids in the presence of flow

**DOI:** 10.1007/s10544-023-00685-9

**Published:** 2023-11-27

**Authors:** Barbara Wirthl, Christina Janko, Stefan Lyer, Bernhard A. Schrefler, Christoph Alexiou, Wolfgang A. Wall

**Affiliations:** 1 Institute for Computational Mechanics, Technical University of Munich, TUM School of Engineering and Design, Department of Engineering Physics & Computation, Garching bei München, Germany; 2 Department of Otorhinolaryngology, Head and Neck Surgery, Section of Experimental Oncology and Nanomedicine (SEON), Else Kröner-Fresenius-Stiftung Professorship, Universitätsklinikum Erlangen, Erlangen, Germany; 3 Department of Otorhinolaryngology, Head and Neck Surgery, Section of Experimental Oncology and Nanomedicine (SEON), Professorship for AI-Guided Nanomaterials within the framework of the Hightech Agenda (HTA) of the Free State of Bavaria, Universitätsklinikum Erlangen, Erlangen, Germany; 4 Department of Civil, Environmental and Architectural Engineering, University of Padua, Padua, Italy; 5 Institute for Advanced Study, Technical University of Munich, Garching bei München, Germany

**Keywords:** Magnetic nanoparticles, Magnetic drug targeting, Multiphase porous media, Tumour-growth model, Cylindrical permanent magnet

## Abstract

**Abstract:**

One of the main challenges in improving the efficacy of conventional chemotherapeutic drugs is that they do not reach the cancer cells at sufficiently high doses while at the same time affecting healthy tissue and causing significant side effects and suffering in cancer patients. To overcome this deficiency, magnetic nanoparticles as transporter systems have emerged as a promising approach to achieve more specific tumour targeting. Drug-loaded magnetic nanoparticles can be directed to the target tissue by applying an external magnetic field. However, the magnetic forces exerted on the nanoparticles fall off rapidly with distance, making the tumour targeting challenging, even more so in the presence of flowing blood or interstitial fluid. We therefore present a computational model of the capturing of magnetic nanoparticles in a test setup: our model includes the flow around the tumour, the magnetic forces that guide the nanoparticles, and the transport within the tumour. We show how a model for the transport of magnetic nanoparticles in an external magnetic field can be integrated with a multiphase tumour model based on the theory of porous media. Our approach based on the underlying physical mechanisms can provide crucial insights into mechanisms that cannot be studied conclusively in experimental research alone. Such a computational model enables an efficient and systematic exploration of the nanoparticle design space, first in a controlled test setup and then in more complex *in vivo* scenarios. As an effective tool for minimising costly trial-and-error design methods, it expedites translation into clinical practice to improve therapeutic outcomes and limit adverse effects for cancer patients.

**Graphic Abstract:**

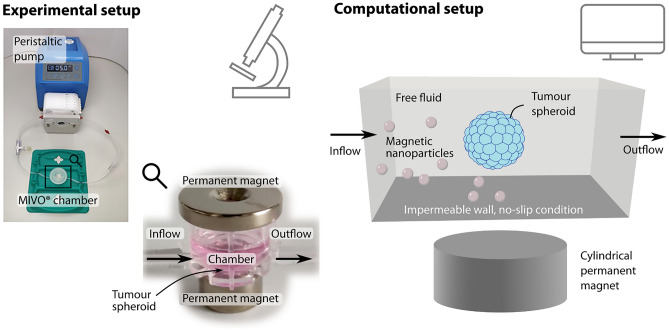

## Introduction

While chemotherapy is one of the most common treatments for cancer, it comes with a significant drawback: the administered drugs do not reach the cancer cells at sufficiently high doses while at the same time attacking healthy tissues. This causes significant side effects and suffering in cancer patients. Magnetic nanoparticles have emerged as a promising approach to overcome this deficiency by achieving more specific tumour targeting. Chemical functionalisation of the nanoparticle surface allows for attaching a chemotherapeutic agent, and an external magnetic field directs the nanoparticles to the target tissue (Flores-Rojas et al. 2022). Such magnetic carriers allow targeting the tumour with enhanced uptake at the target site while reducing the systemic toxicity of the drug (Dobson 2006). For example, Janko et al. (2013) and Tietze et al. (2013) demonstrated that the accumulation of mitoxantrone-loaded magnetic nanoparticles enhanced the anti-tumour efficacy of the drug while sparing the peripheral blood cells.

The unique physical properties of nanoparticles make them such a promising approach. Due to their small size (1 nm to 100 nm), the particles form a single magnetic domain. Therefore, they become highly magnetic in the presence of an external magnetic field but revert to a non-magnetic state when the field is removed, called superparamagnetism (Mody et al. 2013). The nanoparticles are injected intraarterially, and then a magnet placed on the body surface concentrates the nanoparticles at the target site. The translational magnetic force pulling the particles towards the magnet depends on their magnetic properties and the applied magnetic field and field gradients (Barnsley et al. 2015). However, the magnetic field and the field gradient decrease rapidly with increasing distance (Grief and Richardson 2005). Additionally, counteracting hydrodynamic forces further complicate capturing particles from flow, e.g., blood flow or flow in the interstitium. The pressure gradient between the tumour and the surrounding host tissue causes an outward flow of interstitial fluid from the tumour—an additional transport barrier for nanoparticles (Heldin et al. 2004). While nanoparticles have shown great potential for cancer therapy, their transport to the tumour cells is indeed challenging: Dai et al. (2018) showed that less than 14 out of 1 million (0.0014 % injected dose) intravenously administrated nanoparticles coated with cancer-cell recognising ligands actually reached the tumour cells. Our limited understanding of the underlying mechanisms makes it even more difficult to overcome the transport barriers and improve the efficacy of nanoparticle-based drugs (He et al. 2023).

Hence, we need a more detailed understanding of the mechanisms of nanoparticle transport to and in the tumour, combined with effective design strategies, to improve the efficacy of nanoparticle-based drugs.

To achieve this goal, the tumour and its microenvironment must be considered in their entirety. Historically, new anti-cancer drugs and treatment strategies were developed and tested in classical 2D cell cultures. However, 2D cell cultures cannot reproduce the properties of *in vivo* tumours, especially the complex 3D tumour architecture and microenvironment, and the results of such experiments often do not translate to *in vivo* conditions (Friedrich et al. 2009). Therefore, 3D cell cultures, such as multicellular tumour spheroids, have emerged in recent decades (Nunes et al. 2019), and microtechnologies facilitated the controlled, reproducible development of uniform tumour spheroids (Hirschhaeuser et al. 2010). To study the magnetic capturing of nanoparticles in tumour spheroids in the presence of flow, we use a simplified *in vitro* test setup: a tumour spheroid is placed in a flow chamber with a magnet underneath to capture the nanoparticles, as presented by Behr et al. (2022). We retain the essential characteristics and transport barriers of the tumour microenvironment while reducing the complexity of the *in vivo* scenario to a controlled and monitorable setup.

While experimental research is a powerful tool to deepen our understanding of the intricate processes behind cancer, control over the experimental conditions and measurement techniques limit its scope. Additionally, experiments are expensive and time-consuming, and studying all desired configurations and parameters may not be possible. Some processes may even be entirely inaccessible to experimental research. Computational models can offer additional crucial insight and provide systematic nanoparticle design strategies, which avoid conventional trial-and-error approaches—thereby complementing experimental research. Building computational models on first principles leverages our knowledge of the underlying physical mechanisms and enables translation as far as possible. On the way towards a comprehensive computational model for effective nanoparticle-mediated drug design, we start with a model for our experimental test setup, where we can control the environmental conditions and measure the relevant quantities. The potential of our physics-based computational model will provide a stepping stone to *in vivo* scenarios where control and measurement are limited, and ultimately, such an *in silico* tool can accelerate translation into the clinical setting.

In this contribution, we develop a computational model of the transport of magnetic nanoparticles in the experimental test setup described above: we integrate a model for the transport of magnetic nanoparticles in an external magnetic field with a tumour model, which includes the fluid flow. We use a continuum approach to model the nanoparticle transport based on a diffusion-advection equation and a multiphase porous media model for the tumour spheroid and the fluid flow.

Several computational models have previously been developed to study individual aspects of the transport of nanoparticles under the combined effect of flow and magnetic forces in the tumour microenvironment. Chauhan et al. (2012), Welter and Rieger (2013), Cattaneo and Zunino (2014) and Vilanova et al. (2018) developed tumour models which study the interstitial fluid flow in the tumour microenvironment but without including nanoparticle transport. Concerning computational models of tumour spheroids, Deisboeck et al. (2011), Karolak et al. (2018) and Metzcar et al. (2019) comprehensively reviewed the state of the art. Frieboes et al. (2013), Curtis et al. (2015) and Wirthl et al. (2020) presented models of nanoparticle-based cancer therapy limited to the passive transport of nanoparticles without a magnetic field. Furlani and Ng (2006), Furlani and Furlani (2007) and Hewlin and Tindall (2023) studied the capture of magnetic nanoparticles in flow but focused on blood vessels. Detailed computational models which study magnetic nano-drug delivery systems under consideration of transport barriers of the tumour microenvironment include Shamsi et al. (2018) and Rezaeian et al. (2022).

The novelty of our contribution lies in the coupling of fluid flow around and through the tumour spheroid integrated into a theoretically sound and consistent physics-based multiphase porous media model (that also allows the inclusion of many other effects as demonstrated in the past like tumour growth (Sciumè et al. 2012, 2014a, b) and angiogenesis (Kremheller et al. 2018; Kremheller et al. 2019)) together with the transport of magnetic nanoparticles in an external magnetic field, which is evaluated in a numerically efficient way based on analytical expressions for the magnetic field and force. We focus on an *in vitro* test setup in a perfused microfluidic device. Such setups are a powerful tool because of the control over the environmental conditions, and they even replace *in vivo* experiments in preclinical drug testing (Boussommier-Calleja et al. 2016; Adashi et al. 2023). Developing a computational model for such a setup is hence highly relevant to improving the efficacy of nanoparticle-based drugs.

In the remainder of this contribution, we first detail the experimental test setup and the computational model in Section 2. We then present and discuss the results of the computational model in Section 3 and draw a conclusion in Section 4.

## Methods

To be an effective tool for the study of magnetic nanoparticle-mediated drugs, both the experimental test setup and the computational model must incorporate the following essential aspects: the tumour spheroid, the fluid flow around and through the tumour spheroid, and the transport of the magnetic nanoparticles with the flow and guided by the external magnetic field. In the following, we discuss these aspects first for the experimental test setup and then for the computational model.

### Experimental test setup

**Fig. 1:**
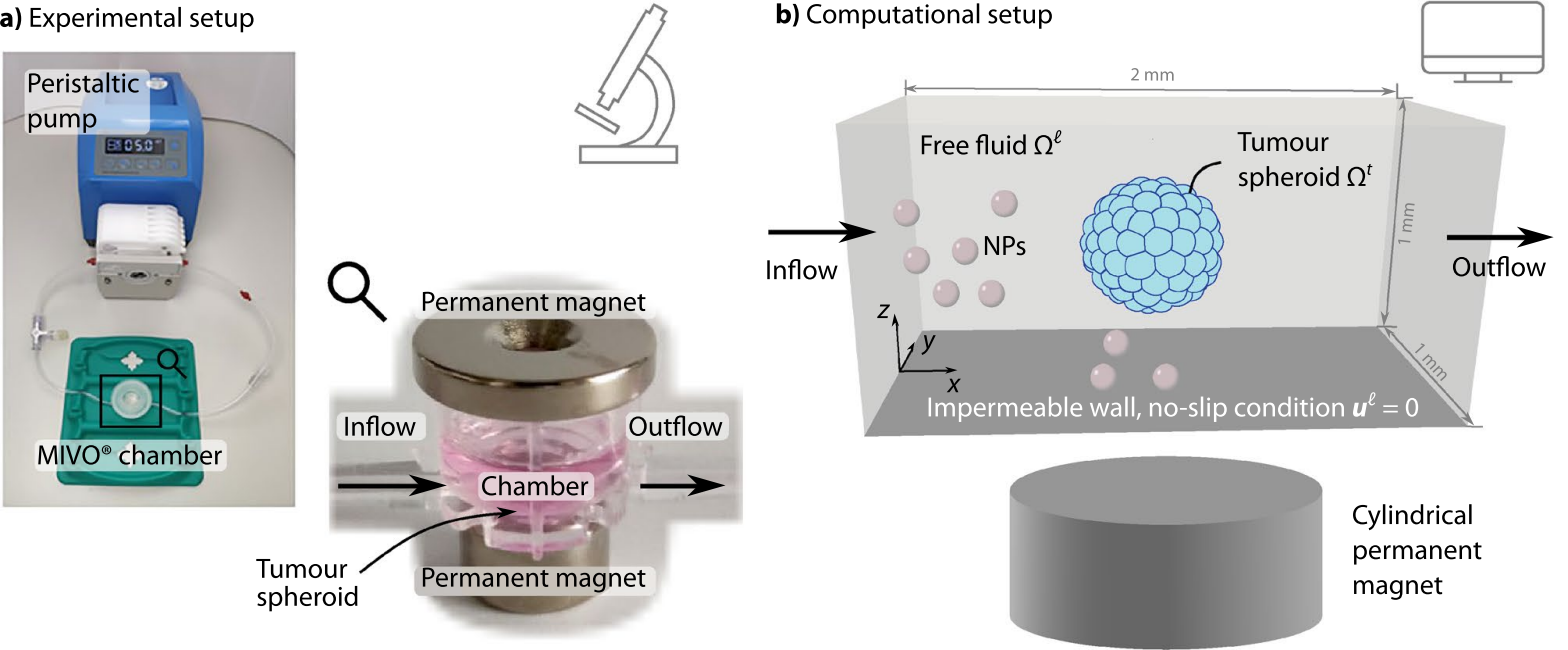
Experimental and computational setup **a** Experimental setup for magnetic accumulations of superparamagnetic iron oxide nanoparticles (SPIONs) in a flow system. The tumour spheroid is placed in a MIVO® chamber connected to a peristaltic pump. Permanent magnets guide the SPIONs to the tumour spheroid. The pictures are adapted from Behr et al. (2022), licensed under CC BY 4.0. **b** Computational setup combining the flow of the free fluid in $\Omega ^\ell$ with a multiphase porous medium for the tumour spheroid in $\Omega ^t$. A cylindrical permanent magnet is positioned below the flow chamber. The nanoparticles (NPs) are transported with the fluid and guided by the magnetic field

In our test setup, we studied the magnetic accumulation of superparamagnetic iron oxide nanoparticles (SPIONs) in a flow chamber. Among the various types of magnetic nanoparticles, SPIONs are the most extensively investigated because of their biocompatibility (Sun et al. 2008). The experimental setup is sketched in Fig. 1a and described in detail in Behr et al. (2022). Therefore, we only give a brief summary below. Tumour spheroids of melanoma cells and fibroblasts were grown for three days. The SPIONs were loaded with a chemotherapeutic drug, in this case, mitoxantrone. To investigate the accumulation of the SPIONs under dynamic conditions, the tumour spheroids were placed in MIVO® single flow chambers without transwell insert (React4Life, Genova, Italy). The flow chamber was perfused using a peristaltic pump, and the SPIONs were injected into the flow. The SPIONs are then accumulated at the tumour spheroid by permanent magnets.

### Computational model

To investigate the transport of magnetic nanoparticles in the test setup described in the previous section, we develop a computational model that specifically tackles the following novel (as compared to our previous work) challenges:

1. Model the free fluid flowing around the tumour spheroid in the flow chamber coupled to the flow in the tumour spheroid.
2. Model the magnetic nanoparticles transported with the fluid and within the tumour spheroid subjected to the force of an external cylindrical magnet.

The computational setup is depicted in Fig. 1b.

We decompose this system into two distinct but fully coupled regions, which we model as a multiphase porous medium: the region of the free fluid $\Omega ^\ell$ and the region of the tumour spheroid $\Omega ^t$. To model the flow in both regions, $\Omega ^\ell$ and $\Omega ^t$, we use a one-domain approach, i.e., we solve the same equation in the entire domain $\Omega = \Omega ^\ell \cup \Omega ^t$: the mass balance equation with a Darcy momentum equation condensed into a single equation. The bottom wall of the domain is impermeable and has a no-slip boundary condition.

The magnetic nanoparticles are dispersed in the fluid and transported by its flow. Additionally, a cylindrical magnet is positioned below the flow chamber and exerts a magnetic force on the nanoparticles. Many publications (Haverkort et al. (2009), Lunnoo and Puangmali (2015), Sharma et al. (2015), Momeni Larimi et al. (2016) and Pálovics and Rencz (2022)) use particle-based approaches for the nanoparticles, where the forces are computed for each individual particle. However, tumour spheroids are on the scale of a few hundred micrometres, while nanoparticles are several orders of magnitude smaller. Investigating the transport of nanoparticles with a particle-based approach at the scale of the tumour spheroid thus involves up to a billion particles—an enormous computational burden (Pálovics and Rencz 2022). But, as we are not interested in the fate of the individual particles, there exist much more efficient alternatives: we use a continuum approach for the nanoparticles, employing a diffusion-advection equation directly at the macroscale. The bottom wall is also impenetrable for the magnetic nanoparticles.

#### Multiphase porous media model for the tumour spheroid and the free fluid

**Fig. 2:**
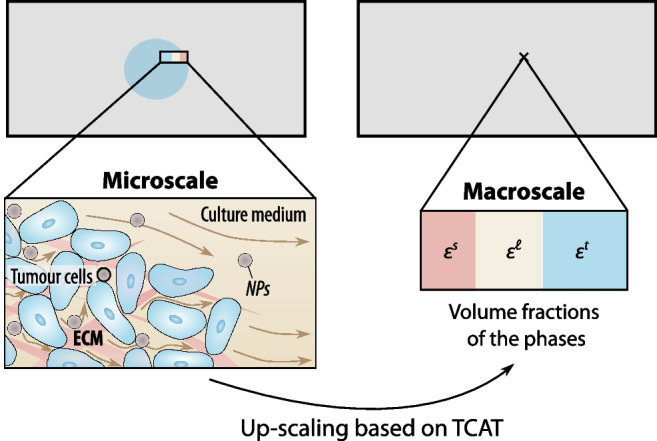
Porous medium with the pore space of the extracellular matrix (ECM) occupied by the tumour cells and the culture medium. The brown arrows indicate the flow of the culture medium, which is transporting the nanoparticles (NP). At the microscale, the different phases can be distinguished (left), while at the macroscale, the phases are described by their volume fractions $\varepsilon ^\alpha$ (right). Up-scaling based on the thermodynamically constrained averaging theory (TCAT) bridges the gap between the two scales

In this study, our model of the tumour spheroid consists of the tumour cells, the extracellular matrix (ECM) and the interstitial fluid (*in vivo*) or the culture medium (in the flow chamber). The ECM is a meshlike structure with voids where the cells are attached or migrate and where the fluid flows. We model the tumour cells as a highly viscous fluid (rather than a solid), as most tumour-growth models do (Sciumè et al. 2013a). The ECM, the tumour cells and the fluid, referred to as phases, together form a porous medium. All phases, including their interfaces, can be distinguished at the microscale (see Fig. 2 left). However, the exact geometry of the ECM is very complex and also not of interest; neither are we interested in the individual cells. Our quantity of interest is the tumour spheroid as a whole, and we therefore describe it at a larger scale, the macroscale. At this scale, the different phases are modelled in an averaged sense and characterised by their volume fractions $\varepsilon ^\alpha$ at a specific point (see Fig. 2 right). To bridge the gap between the microscale and the macroscale, we use the thermodynamically constrained averaging theory (TCAT) (Gray and Miller 2014) to derive the macroscale equations from the microscale equations while retaining a rigorous connection between the two scales (Jackson et al. 2009).

The voids in the ECM constitute the pore space, and the ratio of the volume of the pore space to the total volume is the porosity $\varepsilon$. The fluid phases completely fill and flow in this pore space. In our case, the culture medium and the tumour cells are the fluid phases, denoted by $\ell$ (liquid) and *t* (tumour), respectively. As an adequate assumption in this case, we assume that the ECM does not deform in this study, i.e., the porosity is constant in space and time $\varepsilon = 0.8$ in $\Omega ^t$.

The fluid phases share the pore space of the ECM, and the fraction occupied by each fluid phase is the saturation $S^\alpha$, defined as

1 $$\begin{aligned} S^\alpha = \frac{\varepsilon ^\alpha }{\varepsilon }, \quad \alpha = t,\ell \end{aligned}$$

where $\varepsilon ^\alpha$ is the volume fraction of the fluid phase $\alpha$. We assume the porous medium to be saturated, i.e.,

2 $$\begin{aligned} S^t + S^\ell = 1. \end{aligned}$$

The two fluid phases are governed by the mass balance equation

3 $$\begin{aligned} \rho ^\alpha \varepsilon \frac{\partial S^\alpha }{\partial t} + \varvec{\nabla } \cdot \left( \rho ^\alpha \varepsilon S^\alpha \varvec{u}^\alpha \right) = 0 \end{aligned}$$

where $\rho ^\alpha$ is the density and $\varvec{u}^\alpha$ the velocity of the fluid. The velocity of the fluid phases is defined based on Darcy’s law

4 $$\begin{aligned} \varvec{u}^\alpha = - \frac{1}{\varepsilon S^\alpha } \varvec{K}^\alpha \varvec{\nabla } p^\alpha \end{aligned}$$

with $p^\alpha$ being the pressure of the fluid phase. We define the tensor $\varvec{K}^\alpha$ as the permeability tensor divided by the dynamic viscosity $\mu ^\alpha$ of the fluid phase

5 $$\begin{aligned} \varvec{K}^\alpha = \frac{k_{\textsf {rel}}^\alpha k \varvec{I}}{\mu ^\alpha }, \quad \text {with} \quad k_{\textsf {rel}}^\alpha = \left( S^\alpha \right) ^{A_\alpha }, \end{aligned}$$

where $k_{\textsf {rel}}^\alpha$ is the relative permeability and *k* is the intrinsic permeability of the ECM. The permeability describes how easily a specific fluid flows through the ECM. Since the culture medium and the tumour cells share the same pore space, they interact: the flow of one phase impedes the flow of the other. The relative permeability thus is a function of the corresponding saturation $S^\alpha$ and a model coefficient $A_\alpha$ (Sciumè et al. 2014a).

In this contribution, we employ our previously developed tumour-growth model (see, e.g., Sciumè et al. (2013b) and Kremheller et al. (2018)) to generate a physically plausible initial condition for the tumour spheroid: it results in a saturation of the tumour cell phase of $S^t = 0.8$ and a saturation of the culture medium phase of $S^\ell = 0.2$. We then use these results as an initial condition for studying the transport of magnetic nanoparticles in the flow chamber. The tumour-growth model details can be found in Sciumè et al. (2013b) and Kremheller et al. (2018).

As described above, we use a one-domain approach and solve one equation (in this case Eq. 3) on the entire domain. Outside the tumour spheroid, the culture medium is the only phase, and no ECM is present; thus, $\varepsilon = 1.0 = \text {const.}$ and $S^\ell = 1.0$. In $\Omega ^\ell$, we set the tensor $\varvec{K}^\ell$ and the pressure gradient so that the average velocity of the culture medium is $\varvec{u}^\ell = 0.25 \text { mm s}^{-1}$ based on Eq. 4. We assume the flow to be laminar and the Reynolds number to be small, as is typically the case in microfluidic devices (Brody et al. 1996; Stone et al. 2004).^1^ In addition, we only consider a steady state, i.e., the velocities do not change in time, and neglect body forces, e.g., the gravitational force. At the bottom wall, we apply a no-slip boundary condition, i.e., $\varvec{u}^\ell = 0$.

In sum, employing the multiphase porous media model captures important aspects: the fluid flow around and through the tumour spheroid and the interaction of the flow with the tumour cells. The implications of the fluid flow are crucial both to understand tumour growth better and to improve the design of drug delivery systems (Munson and Shieh 2014; Henke et al. 2020).

In the following, we refer to the flow of the culture medium as the *fluid flow* because we do not further investigate the flow of the tumour cells as the second fluid phase.

#### Transport of the magnetic nanoparticles

We model the transport of the magnetic nanoparticles with a diffusion-advection equation as a continuum approach. The mass fraction $\omega ^{\textsf {NP}\ell }$ of the magnetic nanoparticles in the medium, i.e., the fraction of the mass of the medium that is due to the presence of the nanoparticles (Gray and Miller 2014), is governed by the mass balance equation

6 $$\begin{aligned} \rho ^\ell \frac{\partial (\varepsilon S^\ell \omega ^{\textsf {NP}\ell })}{\partial t} + \varvec{\nabla } \cdot \left( \rho ^\ell \varvec{q}^{\textsf {NP}}\right) = 0, \end{aligned}$$

where $\varvec{q}^{\textsf {NP}}$ is the flux of the magnetic nanoparticles. We assume that no (chemical) reaction within the phase occurs. Further, we here do not investigate the cellular uptake of nanoparticles and intracellular transport; hence, we do not include mass transfer to other phases. The flux is the sum of three transport mechanisms: diffusion, advection with the fluid flow and magnetophoresis (= the motion of magnetic particles in response to an external magnetic field (Ayansiji et al. 2020)), i.e.,

7 $$\begin{aligned} \varvec{q}^{\textsf {NP}}= \varvec{q}^{\textsf {NP}}_{\textsf {diff}} + \varvec{q}^{\textsf {NP}}_{\textsf {adv}} + \varvec{q}^{\textsf {NP}}_{\textsf {mag}}. \end{aligned}$$

Firstly, Fick’s first law describes the diffusive flux as

8 $$\begin{aligned} \varvec{q}^{\textsf {NP}}_{\textsf {diff}} = - \varepsilon S^\ell D^{\textsf {NP}}\varvec{\nabla } \omega ^{\textsf {NP}\ell }, \end{aligned}$$

with the diffusion coefficient $D^{\textsf {NP}}$ and secondly, the advective flux results from the velocity $\varvec{u}^\ell$ of the fluid transporting the nanoparticles, i.e., based on Eq. 4

9 $$\begin{aligned} \varvec{q}^{\textsf {NP}}_{\textsf {adv}} = \omega ^{\textsf {NP}\ell }\varepsilon S^\ell \varvec{u}^\ell = - \omega ^{\textsf {NP}\ell }\varvec{K}^\ell \varvec{\nabla } p^\ell . \end{aligned}$$

Finally, the flux due to magnetophoresis depends on the magnetic force $\varvec{F}_{\textsf {mag}}$, such that

10 $$\begin{aligned} \varvec{q}^{\textsf {NP}}_{\textsf {mag}}= \omega ^{\textsf {NP}\ell }\varepsilon S^\ell \varvec{u}_{\textsf {mag}}= \omega ^{\textsf {NP}\ell }\varvec{\mathcal {M}}^{\textsf {NP}}\varvec{F}_{\textsf {mag}}, \end{aligned}$$

similar to Grief and Richardson (2005) and Furlani and Ng (2006). We here employ the mobility tensor $\varvec{\mathcal {M}}^{\textsf {NP}}$: it relates the applied magnetic force to the resulting velocity of the magnetic nanoparticles, similar to the permeability tensor that relates the pressure gradient to the velocity of the fluid. Classically, the mobility is a scalar defined as

11 $$\begin{aligned} \mathcal {M}^{\textsf {NP}}= \frac{1}{6\pi \mu ^\ell R^{\textsf {NP}}}, \end{aligned}$$

based on Stokes’ law, with $R^{\textsf {NP}}$ being the radius of the nanoparticles and $\mu ^\ell$ the dynamic viscosity (Bird et al. 2002). Using a scalar mobility implies that the velocity $\varvec{u}_{\textsf {mag}}$ is directly proportional to the magnetic force $\varvec{F}_{\textsf {mag}}$. This holds for magnetic particles in the middle of the domain but obviously not for particles close to the impenetrable wall at the bottom. When the magnet is placed below the flow chamber, the magnetic force has a component perpendicular to the wall, which would lead to the nanoparticles penetrating the wall and leaving the domain—which is obviously physically impossible. We therefore use a $3 \times 3$ mobility tensor $\varvec{\mathcal {M}}^{\textsf {NP}}$ with entries only on the main diagonal, as presented in Wirthl et al. (2023): inside the domain, the mobility is the scalar mobility given in Eq. 11, and at the impenetrable wall, the mobility perpendicular to the wall is zero, i.e., $\mathcal {M}_{z} = 0$, so the nanoparticles cannot penetrate the wall. To avoid numerical instabilities, we set a smooth transition between the mobility inside the domain and the mobility at the wall.

We further introduce a relative mobility $m_{\textsf {rel}}^\ell$ similar to the relative permeability $k_{\textsf {rel}}^\ell$ in Eq. 5. All diagonal entries of the mobility tensor inside the domain are given by

12 $$\begin{aligned} \mathcal {M}_{x} = \mathcal {M}_{y} = \mathcal {M}_{z} = \frac{m_{\textsf {rel}}^\ell }{6\pi \mu ^\ell R^{\textsf {NP}}} \quad \text {with} \quad m_{\textsf {rel}}^\ell = \left( S^\ell \right) ^{A_\ell }. \end{aligned}$$

The advantages of our approach based on the mobility tensor are twofold. Firstly, we include a simple approach to model nanoparticle accumulation at the wall—as opposed to Furlani and Furlani (2007) and Roa-Barrantes and Rodriguez Patarroyo (2022) where the boundary condition at the wall is unclear. Secondly, the relative mobility allows us to consider the interaction of the nanoparticles with other phases—which is especially relevant for the interaction with the ECM (He et al. 2023).

In sum, the mass balance equation for the magnetic nanoparticles is given by

13 $$\begin{aligned} &{} \rho ^\ell \varepsilon S^\ell \frac{\partial \omega ^{\textsf {NP}\ell }}{\partial t} - \varvec{\nabla } \cdot \left( \rho ^\ell \varepsilon S^\ell D^{\textsf {NP}}\varvec{\nabla } \omega ^{\textsf {NP}\ell }\right) \\ &{} - \rho ^\ell \varvec{K}^\ell \varvec{\nabla } p^\ell \cdot \varvec{\nabla } \omega ^{\textsf {NP}\ell }+ \varvec{\nabla } \cdot \left( \rho ^\ell \omega ^{\textsf {NP}\ell }\varvec{\mathcal {M}}^{\textsf {NP}}\, \varvec{F}_{\textsf {mag}}\, \right) = 0, \end{aligned}$$

which is a Smoluchowski advection–diffusion equation (Smoluchowski 1915).

#### Magnetic force on the nanoparticles

When the (superparamagnetic) nanoparticles are subjected to the external magnetic field $\varvec{H}$ of the cylindrical permanent magnet, they magnetise, inducing a magnetic dipole in the particles. The magnetic force $\varvec{F}_{\textsf {mag}}$ acting on the magnetic nanoparticles depends on the applied magnetic field as well as on the magnetic response of the particles. It is given by

14 $$\begin{aligned} \varvec{F}_{\textsf {mag}}= \mu _0 V^{\textsf {NP}}f(\varvec{H}) \left( \varvec{H} \cdot \varvec{\nabla } \right) \varvec{H}, \end{aligned}$$

where $\mu _0$ is the vacuum magnetic permeability, $V^{\textsf {NP}}$ is the volume of the nanoparticles and $f(\varvec{H})$ is the magnetisation model of the particles (Pankhurst et al. 2003; Furlani and Ng 2006). We assume the inter-particle distance to be large enough that inter-particle forces are negligible (Furlani and Ng 2006; Keaveny and Maxey 2008; Han et al. 2010; Khashan et al. 2011; Woińska et al. 2013; Barrera et al. 2021). Outside the magnet, the magnetic flux density $\varvec{B}$ (also called B-field) is related to the magnetic field $\varvec{H}$ by $\varvec{B} = \mu _0 \varvec{H}$.

To evaluate Eq. 14, we need to compute the magnetic field $\varvec{H}$ and its derivatives. Analytic expressions are only well-known for classic textbook cases, such as the magnetic field of a straight wire or a solenoid. Otherwise, the magnetic field must be computed based on numerically solving Maxwell’s equations. For the particular case of a cylindrical magnet with a finite length and longitudinal magnetisation, Derby and Olbert (2010) and Caciagli et al. (2018) derived an analytic expression for the magnetic field, which we restate in the following.

The magnetic field $\varvec{H}$ in cylindrical coordinates $(\rho , \phi , z)$ is given by

15 $$\begin{aligned} H_\rho (\rho , z)&= \frac{M_s R_{\textsf {mag}}}{\pi } \left[ \alpha _+ P_1(k_+) - \alpha _- P_1(k_-) \right] \end{aligned}$$

16 $$\begin{aligned} H_z(\rho , z)&= \frac{M_s R_{\textsf {mag}}}{\pi (\rho + R_{\textsf {mag}})} \left[ \beta _+ P_2(k_+) - \beta _- P_2(k_-) \right] \end{aligned}$$

and $H_\phi = 0$ due to the radial symmetry. Further, $M_s$ is the magnetisation of the magnet and $R_{\textsf {mag}}$ its radius. The two auxiliary functions $P_1$ and $P_2$ are defined based on the complete elliptic integrals of the first, second and third kind $\mathcal {K}$, $\mathcal {E}$ and $\Pi$ as

17 $$\begin{aligned} P_1(k)&= \mathcal {K}\left( 1 - k^2\right) - \frac{2}{1 - k^2} \left[ \mathcal {K}\left( 1 - k^2\right) - \mathcal {E}\left( 1 - k^2\right) \right] \end{aligned}$$

18 $$\begin{aligned} \begin{aligned} P_2(k)&= -\frac{\gamma }{1 - \gamma ^2} \left[ \Pi \left( 1 - \gamma ^2, 1 - k^2\right) - \mathcal {K}\left( 1 - k^2\right) \right] \\&\quad - \frac{1}{1 - \gamma ^2} \left[ \gamma ^2 \Pi \left( 1 - \gamma ^2, 1 - k^2\right) - \mathcal {K}\left( 1 - k^2\right) \right] . \end{aligned} \end{aligned}$$

The auxiliary parameters are defined as

$$\begin{aligned} \rho _{\pm }&= R_{\textsf {mag}}\pm \rho , \quad \zeta _{\pm } = \frac{L}{2} \pm z, \quad \alpha _{\pm } = \frac{1}{\sqrt{ \zeta _{\pm }^2 + \rho _{+}^2}}, \\ \beta _{\pm }&= \zeta _{\pm } \alpha _{\pm }, \quad \gamma = \frac{\rho - R_{\textsf {mag}}}{\rho _+}, \quad k_{\pm } = \sqrt{\frac{\zeta _{\pm }^2 + \rho _{-}^2}{\zeta _{\pm }^2 + \rho _{+}^2}}. \end{aligned}$$

based on Derby and Olbert (2010) and Caciagli et al. (2018). The elliptic integrals can be evaluated efficiently based on Carlsons’s functions (Carlson 1979; Carlson and Notis 1981), and the algorithms and source code are available in *Numerical Recipes* (Press et al. 2007). We thus have an analytic expression for the magnetic field $\varvec{H}$, which we can use to compute the magnetic force $\varvec{F}_{\textsf {mag}}$ in Eq. 14.

For an analytic expression of the magnetic force $\varvec{F}_{\textsf {mag}}$, we additionally need to evaluate the first derivatives of the magnetic field $\varvec{H}$: the first derivatives of all three elliptic integrals are known analytically and can again be represented in closed form through the elliptic integrals. Therefore, for the particular case of a cylindrical magnet with a finite length and longitudinal magnetisation, an analytic expression for Eq. 14 is available, as presented in Wirthl et al. (2023). Because of the lengthy expressions, we do not repeat them here but refer the interested reader to Wirthl et al. (2023).

Due to their small size, the nanoparticles are superparamagnetic, i.e., they possess a high magnetic susceptibility $\chi ^{\textsf {NP}}\gg 1$ (Sun et al. 2008; McNamara and Tofail 2017). We use a linear magnetisation model with saturation given by

19 $$\begin{aligned} f(\varvec{H}) = {\left\{ \begin{array}{ll} \; 3 &{}\text {if }|\varvec{H}| < \frac{1}{3}M_{\textsf {sp}} \\ \frac{M_{\textsf {sp}}}{|\varvec{H}|} &{} \text {if }|\varvec{H}| \ge \frac{1}{3}M_{\textsf {sp}} \end{array}\right. } \end{aligned}$$

with the saturation magnetisation $M_{\textsf {sp}}$ of the nanoparticles (Furlani and Ng 2006; Hallmark et al. 2019), based on the experimental results of Takayasu et al. (1983). Below saturation, the magnetisation is directly proportional to the applied magnetic field $\varvec{H}$, and above saturation, the magnetisation is equal to the saturation magnetisation $M_{\textsf {sp}}$ and aligned with the applied magnetic field $\varvec{H}$.

Having an analytic expression has the great advantage that the magnetic force $\varvec{F}_{\textsf {mag}}$ can be evaluated at all coordinates with minimal computational effort compared to numerically solving Maxwell’s equations.

#### Computational solution approach

For the tumour cell phase, we solve Eq. 3 for the pressure of the tumour cell phase $p^t$. As the second governing equation for the fluid phases, we do not solve Eq. 3 for the culture medium phase directly but rather sum up the mass balance equations of the two fluid phases and solve the resulting equation for the pressure $p^\ell$, given by

20 $$\begin{aligned} {\varvec{\nabla } \cdot \left( \frac{k_{\textsf {rel}}^t k \varvec{I}}{\mu ^t} \varvec{\nabla } p^t \right) + \varvec{\nabla } \cdot \left( \frac{k_{\textsf {rel}}^\ell k \varvec{I}}{\mu ^\ell } \varvec{\nabla } p^\ell \right) = 0. }\end{aligned}$$

This summed-up equation includes several simplifications, e.g., invoking the sum of saturations Eq. 2. For further details, see Sciumè et al. (2014b).

To solve the governing equations Eqs. (3), (13) and (20) in space and time, we use the standard Galerkin procedure to obtain the weak form of the equations and then discretise the equations in space and time; for the discretisation in space, we employ the finite element method and the backwards Euler method for the discretisation in time. The system of equations is strongly coupled, and we apply a monolithic solution algorithm with a single Newton–Raphson loop per time step. The resulting linear system of equations has a block structure, and we solve it using a generalised minimal residual method (GMRES) iterative solver with a preconditioner based on a block Gauss–Seidel (BGS) method combined with an algebraic multigrid (AMG) method (for further details see, e.g., Verdugo and Wall (2016) and Verdugo et al. (2017) and Fang et al. (2019)). As a computational framework, we use the in-house parallel multiphysics research code BACI (BACI 2023).

In our case, the convective terms dominate the Smoluchowski advection–diffusion equation, which causes numerical instabilities when using the standard Galerkin procedure. To overcome this issue, we use the streamline-upwind Petrov–Galerkin (SUPG) method (Brooks and Hughes 1982) to stabilise both convective terms, and we choose the stabilisation parameter $\tau$ as proposed by Codina (2002).

## Numerical results and discussion

### Computational setup

Figure 1b sketches the computational setup. We investigate four different configurations of the tumour spheroid: two different tumour spheroid sizes ($R_{\textsf {small}}^t \approx {200\,\mathrm{\upmu \text {m}}}$ and $R_{\textsf {large}}^t \approx {340\,\mathrm{\upmu \text {m}}}$) and two different positions (centred or lying at the bottom of the chamber). The tumour spheroid placed at the bottom of the chamber mimics our experimental test setup. The configurations with the tumour spheroid in the centre of the chamber assumes that the tumour spheroid is placed into an insert, fitting the flow chamber (Marzagalli et al. 2022).

The porosity is 1.0 outside and 0.8 inside the tumour spheroid (Sciumè et al. 2014b). The culture medium only occupies a volume fraction of $\varepsilon ^\ell = \varepsilon S^\ell = 0.12$ inside the tumour spheroid, with the ECM and tumour cells sharing in remaining volume. This sharp gradients in the primary variables of the fluid field at the edge of the tumour spheroid require a fine discretisation of the domain. The entire computational domain of ${2\,\mathrm{\text {m}\text {m}}} \times {1\,\mathrm{\text {m}\text {m}}} \times {1\,\mathrm{\text {m}\text {m}}}$ is discretised with $\sim 2 \times 10^6$ linear hexahedral elements, with all elements being perfect cubes of equal size. The time step is $\Delta t = {1\,\mathrm{\text {s}}}$, and the simulation time is 100 s.

Concerning the boundary conditions of the fluid field, we apply the pressure $p^\ell$ as Dirichlet boundary condition at the inflow and outflow such that based on Eq. (4), this pressure difference together with the tensor $\varvec{K}$ results in an average velocity of $\varvec{u}^\ell = 0.25 \text { mm s}^{-1}$ in $\Omega ^\ell$. At the bottom wall, we apply a no-slip boundary condition, i.e., $\varvec{u}^\ell = 0$. As explained above, the fluid flow in $\Omega ^\ell$ is fully coupled to the fluid flow in $\Omega ^t$.

Concerning the boundary conditions of the magnetic nanoparticles, at the inflow, we prescribe the mass fraction of nanoparticles $\omega ^{\textsf {NP}\ell }$ as Dirichlet boundary condition given by a sigmoid function with the value of 0 at the lower third of the inflow boundary and the value of $1 \times 10^{-6}$ at the upper part of the inflow boundary. The bottom boundary is impenetrable for the nanoparticles, i.e., the mobility perpendicular to the boundary is zero $\mathcal {M}_{z} = 0$, so the nanoparticles cannot penetrate the wall.

Concerning the magnetic field and force, we assume a cylindrical magnet with a length of $L_{\textsf {mag}}= {1\,\mathrm{\text {m}\text {m}}}$ and a radius of $R_{\textsf {mag}}= {0.5\,\mathrm{\text {m}\text {m}}}$. Both the magnetic field and the magnetic force are computed based on the analytic expressions presented in Eqs. (15) and (16) and Wirthl et al. (2023), respectively. Therefore, no further boundary conditions are required—as opposed to solving Maxwell’s equations numerically.

Table 1 summarises the employed parameters for the magnetic nanoparticles, the cylindrical magnet and the fluid phases: all values are based on experimental results or previous computational studies in the literature.

**Table 1:** Parameters for the magnetic nanoparticles, the cylindrical magnet and the fluid phases

**Symbol**	**Parameter**	**Value**	**Units**	**Ref.**
*Magnetic nanoparticles*				
$R^{\textsf {NP}}$	Radius of the nanoparticles	100	nm	Furlani and Ng (2006)
$D^{\textsf {NP}}$	Diffusion coefficient	$2.5 \times 10^{-10}$	$\text {m}^2 \text { s}^{-1}$	Lahonian (2013)
$M_{\textsf {sp}}$	Saturation magnetisation	$4.78 \times 10^5$	A m^-1^	Furlani and Ng (2006)
*Cylindrical magnet*				
$L_{\textsf {mag}}$	Length of the magnet	1	mm	Assumed
$R_{\textsf {mag}}$	Radius of the magnet	0.5	mm	Assumed
$M_s$	Magnetisation of the magnet	${1 \times 10^6}$	A m^-1^	Furlani and Ng (2006)
$\mu _0$	Magnetic (vacuum) permeability	${1.25663706212 \times 10^{-6}}$	N A^-2^	Physical constant
*Fluid phases*				
$\rho ^\ell$, $\rho ^t$	Density of the medium and the cells	${1 \times 10^3}$	kg m^-3^	Known
$\mu ^\ell$	Dynamic viscosity of the medium	${1 \times 10^{-3}}$	Pa s	Known
$\mu ^t$	Dynamic viscosity of the cells	20	Pa s	Sciumè et al. (2014b)
*k*	Intrinsic permeability of the ECM	${1 \times 10^{-9}}$	mm^2^	Hervas-Raluy et al. (2023)
$\varepsilon$	Porosity of the ECM	0.8	-	Sciumè et al. (2014b)
$A_\ell$	Relative permeability exponent of the medium	4	−	Sciumè et al. (2014a)
$A_t$	Relative permeability exponent of the cells	2	−	Kremheller et al. (2018)

### Fluid flow

**Fig. 3:**
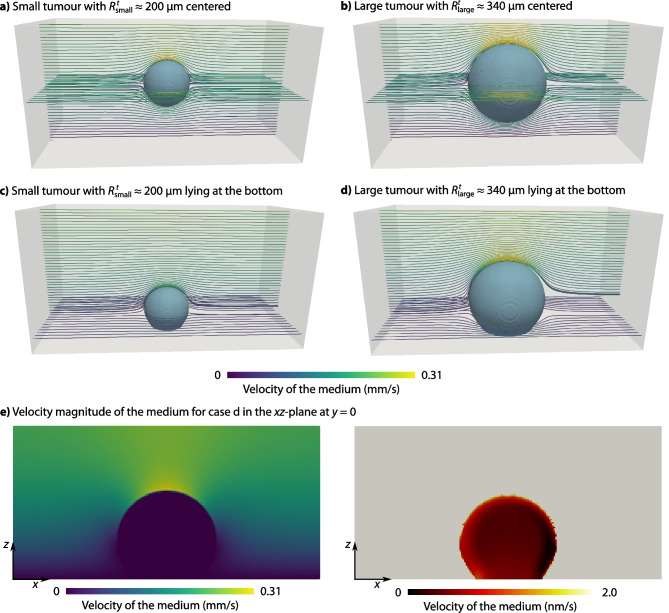
Velocities in the flow chamber for different tumour spheroid sizes and positions **a** Small tumour spheroid centred in the flow chamber. **b** Large tumour spheroid centred in the flow chamber. **c** Small tumour spheroid lying at the bottom of the flow chamber. **d** Large tumour spheroid lying at the bottom of the flow chamber. **e** Velocity magnitude for the large tumour spheroid lying at the bottom of the flow chamber (case d): velocity magnitude in the free fluid (left) in mm/s and in the tumour spheroid (right) in nm/s

We first analyse the fluid flow around and through the tumour spheroid for the four different configurations. Figure 3 depicts the results: the flow around the tumour spheroid resembles the classical Stokes flow around a sphere. The velocity is zero at the impenetrable wall at the bottom of the flow chamber, and the highest velocities occur at the top edge of the tumour spheroid. The bulk of the fluid flows around the tumour, and the velocities inside the tumour are much smaller. Nevertheless, the fluid in the tumour is not stagnant: the fluid velocities are of the order of nm s^-1^.

We employ a one-domain approach due to its simplicity while retaining essential physics. If more complex flow patterns around the tumour spheroid are of interest, such as transitional flow with vortices or even turbulent flow, one can solve the Navier–Stokes equations in $\Omega ^\ell$. Based on a two-domain approach, the free fluid is described by the Navier–Stokes equations and coupled to the solid structure or the porous medium via interface conditions, see for example Girault et al. (2013), Coroneo et al. (2014) or Ager et al. (2019).

### Nanoparticle distribution

We now investigate the distribution of the nanoparticles for the four different configurations. For all configurations, the nanoparticles are injected in the upper half of the inflow boundary with a mass fraction of $\omega ^{\textsf {NP}\ell } = {1.0 \times 10^{-6}}{}$. The cylindrical magnet has a radius of $R_{\textsf {mag}}= {0.5\,\mathrm{\text {m}\text {m}}}$ and a length of $L_{\textsf {mag}}= {1\,\mathrm{\text {m}\text {m}}}$. The centre of the magnet is positioned at $x = {1.0\,\mathrm{\text {m}\text {m}}}$, centred in the y-direction with a vertical distance of ${0.25\,\mathrm{\text {m}\text {m}}}$ to the bottom of the domain.

**Fig. 4:**
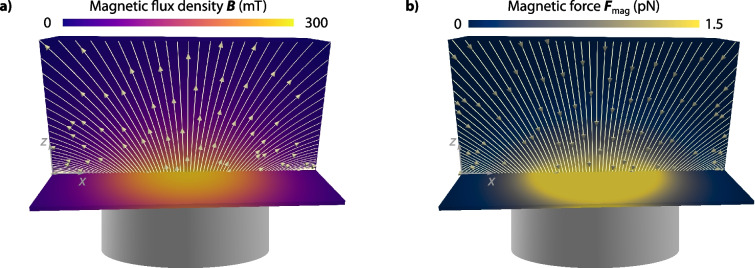
Magnetic flux density and force for the cylindrical magnet vertically positioned below the flow chamber at a distance of ${0.25\,\mathrm{\text {m}\text {m}}}$ from the bottom of the domain. The magnet has a radius of 0.5 mm and a length of 1 mm. **a** Magnetic flux density $\varvec{B}$. **b** Magnetic force $\varvec{F}_{\textsf {mag}}$

The resulting magnetic flux density $\varvec{B}$ and magnetic force $\varvec{F}_{\textsf {mag}}$ in the computational domain are presented in Fig. 4. Both the magnetic flux density and the magnetic force are highest directly at the edge of the magnet but rapidly decrease with distance. The smaller the magnet, the harder it is to capture nanoparticles at the top of the domain. The maximum magnetic flux density is $|\varvec{B}|_{\textsf {max}} = \mu _0 | \varvec{H}|_{\textsf {max}} = {300\,\mathrm{\text {m}\text {T}}}$, which is of the same order of magnitude as the magnetic flux density in our experimental setup (Behr et al. 2022). The maximum magnetic force in the domain is of the order of pN, which is larger than the values estimated in Pálovics and Rencz (2022) but on a similar order of magnitude.

**Fig. 5:**
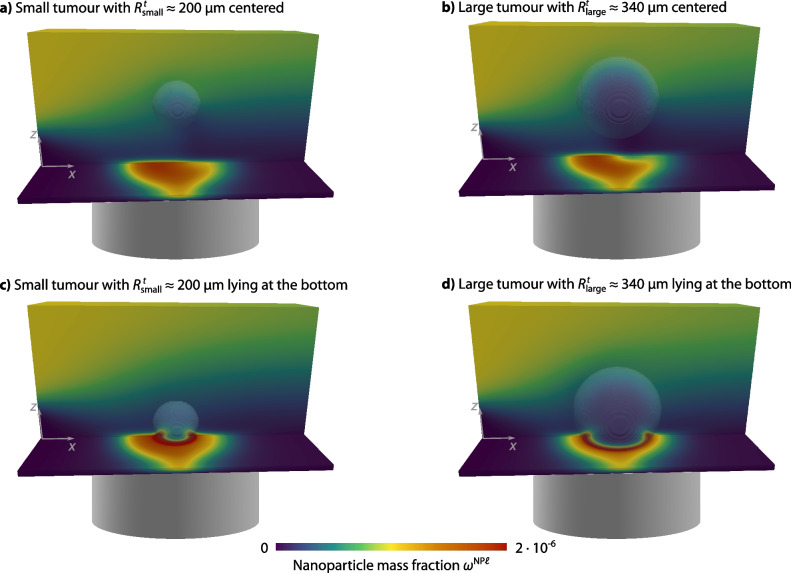
Results for the nanoparticle mass fractions $\omega ^{\textsf {NP}\ell }$ at $t = {20\,\mathrm{\text {s}}}$ for different tumour spheroid sizes and positions

Figure 5 depicts the nanoparticle distribution at $t = {20\,\mathrm{\text {s}}}$ for the four different configurations. The nanoparticles accumulate just above where the magnet is positioned. More nanoparticles accumulate at the left side of the magnet due to the flow direction and the fact that the velocity in the lower part of the domain is decreasing because of the no-slip condition at the bottom wall. In cases c and d, where the tumour spheroid is positioned at the bottom of the domain, the nanoparticles form a ring-like structure around the edge of the tumour spheroid.

The results in Fig. 5 further show that the nanoparticles have not yet fully penetrated the tumour spheroid after 20 s but are located close to the surface, similar to what has been observed experimentally (Tchoryk et al. 2019; Ahmed-Cox et al. 2022). The penetration of the nanoparticles into the tumour spheroid is a complex process, which we do not study in further detail here. Dai et al. (2018) quantified that only 0.0014% of the intravenously injected nanoparticles reach the tumour cells, and He et al. (2023) discussed the ECM as the main steric obstacle for nanoparticle diffusion in the tumour. However, the underlying mechanisms remain largely unexplored. Experimental measurements of the diffusion coefficient of nanoparticles in the tumour vary significantly and indicate that the diffusion coefficient depends on the physicochemical properties of the nanoparticles, the tumour type, and the tumour microenvironment (Dai et al. 2018). A more detailed study of nanoparticle transport in the tumour spheroid is consequently required, both experimentally and computationally, to overcome this transport barrier and improve the efficacy of nanoparticle-based drug delivery systems.

We here only consider the force exerted by the external magnetic field. However, the magnetised nanoparticles also exert forces on each other when they are close. According to Furlani and Ng (2006), Keaveny and Maxey (2008), Han et al. (2010), Khashan et al. (2011), Woińska et al. (2013) and Barrera et al. (2021), the magnetic inter-particle forces are negligible when the nanoparticles have a distance of more than three particle diameters, which we assume to be the case here and thus neglect the inter-particle forces, similar to Boutopoulos et al. (2020). Additionally, we assume that the nanoparticles have a surface coating that stabilises them against aggregation due to inter-particle surface interaction (Gutiérrez et al. 2019). However, nanoparticles are known to form aggregates, e.g., chains (Pálovics et al. 2020), and in such cases, the inter-particle forces indeed play a significant role. In this context, Cregg et al. (2009, 2010) studied nanoparticle agglomeration with a particle-based approach, including the inter-particle forces for a small number of particles (up to 25). By contrast, Pálovics et al. (2020) presented a continuum model capable of modelling the aggregation of the nanoparticles: they first simulated the aggregate formation at the microscale based on a discrete particle method and then transferred the results to the continuum approach at the macroscale by adapting the viscosity.

We here employ an analytical expression for the magnetic force of a cylindrical magnet of finite length. Furlani and Ng (2006, 2007) and Hewlin and Tindall (2023) assumed the cylindrical magnet to be infinitely long. This however does not allow positioning the magnet perpendicular to the domain (with the magnet axis parallel to the *z*-axis) as we do here. Our approach enables arbitrary length and diameter of the magnet and an arbitrary position of the magnet, thus allowing for a more realistic and flexible simulation of the experimental setup.

Finally, we only study a simplified model in the experimental test setup and the computational model: we consider the tumour spheroid with the ECM and the flow in the flow chamber, but such a setup does not include the blood vessels and the surrounding tissue, as we did in Wirthl et al. (2020) for a different context. Appropriately including all relevant aspects of the tumour environment is crucial for translating the results to *in vivo* scenarios and clinical practice. Accordingly, Stillman et al. (2020) argued that circulation and extravasation are major transport barriers, which *in silco* models should include. The approach we present here is readily extendable to the vascular version of our tumour-growth model, which includes the vasculature, angiogenesis, and the surrounding host tissue (Kremheller et al. 2018; Kremheller et al. 2019; Wirthl et al. 2020; Kremheller et al. 2021). This then allows for integrating the results of magnetic nanoparticle capture in blood vessels (Furlani and Ng 2006; Furlani and Furlani 2007; Hewlin and Tindall 2023) with the results of nanoparticle transport in the tumour spheroid presented here.

## Conclusion

Motivated by a recent experimental test setup (Behr et al. 2022), we presented a computational model for the magnetic capture of nanoparticles in a flow chamber with a tumour spheroid. Our continuum approach for the transport of the nanoparticles based on the Smoluchowski advection–diffusion equation includes the advection by the fluid flow and the magnetophoresis by the external magnetic field. Based on a multiphase porous media approach, our model further couples the flow in the flow chamber to the flow in the tumour spheroid. The magnetic force on the nanoparticles is efficiently calculated using analytical expressions for the magnetic field and magnetic force of a finite-length cylindrical magnet. Investigating the capturing of magnetic nanoparticles in a controlled flow environment, both *in vitro* and *in silico*, forms the basis for further studies in more complex scenarios, e.g., in a vascular *in vivo* model.

Developing a comprehensive *in silico* model will enable a fast and systematic exploration of the nanoparticle design space, which is impossible in experimental research alone: this reduces the number of experiments required to the most promising candidates, bypassing costly and time-consuming trial-and-error design methods (Karolak et al. 2018; Stillman et al. 2020). A collaboration between experimentalists, computational modellers, and clinicians will allow us to build an integrated framework for drug development and accelerate the translation of the results into clinical practice.

## Data Availability

Data generated during this study is available from the authors upon reasonable request.

## Footnotes

1 Given a domain width of $w = {1\,\mathrm{\text {m}\text {m}}}$ and assuming an average flow velocity of $u^\ell = 0.25 \text {mm s}^{-1}$ results in a Reynolds number of $\text {Re} = \rho ^\ell u^\ell w / \mu ^\ell = 0.25 < 1$.
